# Interferon-alpha or -beta facilitates SARS-CoV-2 pulmonary vascular infection by inducing ACE2

**DOI:** 10.1007/s10456-021-09823-4

**Published:** 2021-10-29

**Authors:** Timothy Klouda, Yuan Hao, Hyunbum Kim, Jiwon Kim, Judith Olejnik, Adam J. Hume, Sowntharya Ayyappan, Xuechong Hong, Juan Melero-Martin, Yinshan Fang, Qiong Wang, Xiaobo Zhou, Elke Mühlberger, Hongpeng Jia, Robert F. Padera, Benjamin A. Raby, Ke Yuan

**Affiliations:** 1 Division of Pulmonary Medicine, Boston Children’s Hospital and Harvard Medical School, Boston, MA 02115 USA; 2 Department of Microbiology, Boston University School of Medicine, Boston, MA 02118 USA; 3 National Emerging Infectious Diseases Laboratories, Boston University, Boston, MA 02118 USA; 4 Department of Cardiac Surgery, Boston Children’s Hospital and Harvard Medical School, Boston, MA 02115 USA; 5 Center for Human Development and Division of Digestive and Liver Disease, Department of Medicine, Columbia University Medical Center, New York, NY 10032 USA; 6 Division of Pediatric Surgery, Department of Surgery, The Johns Hopkins University School of Medicine, Baltimore, MD 21205 USA; 7 Division of Pulmonary and Critical Care Medicine, Channing Division of Network Medicine, Brigham and Women’s Hospital, Harvard Medical School, Boston, MA 02115 USA; 8 Department of Pathology, Brigham and Women’s Hospital, Harvard Medical School, Boston, MA 02115 USA

**Keywords:** Endothelial, ACE2, Interferon, SARS-CoV-2, COVID-19

## Abstract

Severe viral pneumonia caused by the severe acute respiratory syndrome coronavirus 2 (SARS-CoV-2) is characterized by a hyperinflammatory state typified by elevated circulating pro-inflammatory cytokines, frequently leading to potentially lethal vascular complications including thromboembolism, disseminated intracellular coagulopathy and vasculitis. Though endothelial infection and subsequent endothelial damage have been described in patients with fatal COVID-19, the mechanism by which this occurs remains elusive, particularly given that, under naïve conditions, pulmonary endothelial cells demonstrate minimal cell surface expression of the SARS-CoV-2 binding receptor ACE2. Herein we describe SARS-CoV-2 infection of the pulmonary endothelium in postmortem lung samples from individuals who died of COVID-19, demonstrating both heterogeneous ACE2 expression and endothelial damage. In primary endothelial cell cultures, we show that SARS-CoV-2 infection is dependent on the induction of ACE2 protein expression and that this process is facilitated by type 1 interferon-alpha (IFNα) or -beta(β)—two of the main anti-viral cytokines induced in severe SARS-CoV-2 infection—but not significantly by other cytokines (including interleukin 6 and interferon γ/λ). Our findings suggest that the stereotypical anti-viral interferon response may paradoxically facilitate the propagation of COVID-19 from the respiratory epithelium to the vasculature, raising concerns regarding the use of exogenous IFNα/β in the treatment of patients with COVID-19.

**Supplementary Information:**

The online version contains supplementary material available at 10.1007/s10456-021-09823-4.

## Introduction

More than 244 million severe acute respiratory syndrome coronavirus 2 (SARS-CoV-2) infections have been confirmed in the one year since its identification. Though the majority of patients survive, with disease limited to mild-to-moderate respiratory involvement, the virus has already claimed more than 4 million lives, largely a consequence of severe pneumonia, acute respiratory distress syndrome (ARDS) and respiratory failure [1]. Though diffuse alveolar damage is a nearly ubiquitous finding in these cases, physiologic evidence of pulmonary vascular dysfunction is frequently observed, and a subset of patients (particularly those with hypertension, diabetes or obesity) develop extra-pulmonary vascular complication—thrombosis, thromboembolism, coagulopathy—with a five to tenfold higher mortality rate [2–6]. Autopsy studies have reported widespread thrombosis with microangiopathy and alveolar capillary microthrombi throughout the pulmonary vasculature [7]. In addition, the frequent observations in severe COVID-19 of marked elevations of plasma von Willebrand Factor (vWF), P-selectin, D-dimers and other coagulation activating factors provide indirect evidence that endothelial damage and the development of vasculopathy as a pivotal determinant of clinical outcome in many patients.

Our study identified the co-presence of SARS-CoV-2 nucleoprotein (N) and Angiotensin-converting enzyme 2 (ACE2) receptor on the pulmonary vascular endothelium in postmortem COVID-19 patient samples. Moreover, the vascular distribution of endothelial viral infection (positive anti-SARS-CoV-2 N staining) and disrupted endothelial structure were observed mostly in larger muscularized arteries and veins (diameter > 100 µm) in four out of six samples. Importantly, we also demonstrated that pro-inflammatory IFNα or β induced ACE2 on primary human pulmonary endothelial cell cultures and showed for the first time that primary endothelial cells were successfully infected with wild type SARS-CoV-2 or transduced by SARS-CoV-2 spike protein pseudotyped HIV viruses. In addition, our study suggested that IFNα or β induced functional ACE2, rather than the proposed non-functional delta (d) ACE2. Our work proposed the critical role of ACE2 upregulation on pulmonary endothelial cells by IFNα or β contributed to the understanding of COVID-19 associated vasculopathy, aiding future research on characterizing SARS-CoV-2 infection in cardiovascular biology. Translationally, our findings also raised concerns about using exogenous IFNα or β treaments in patients with severe COVID-19.

## Results

### Viral staining was positive mostly in larger muscularized arteries and veins

**Fig. 1:**
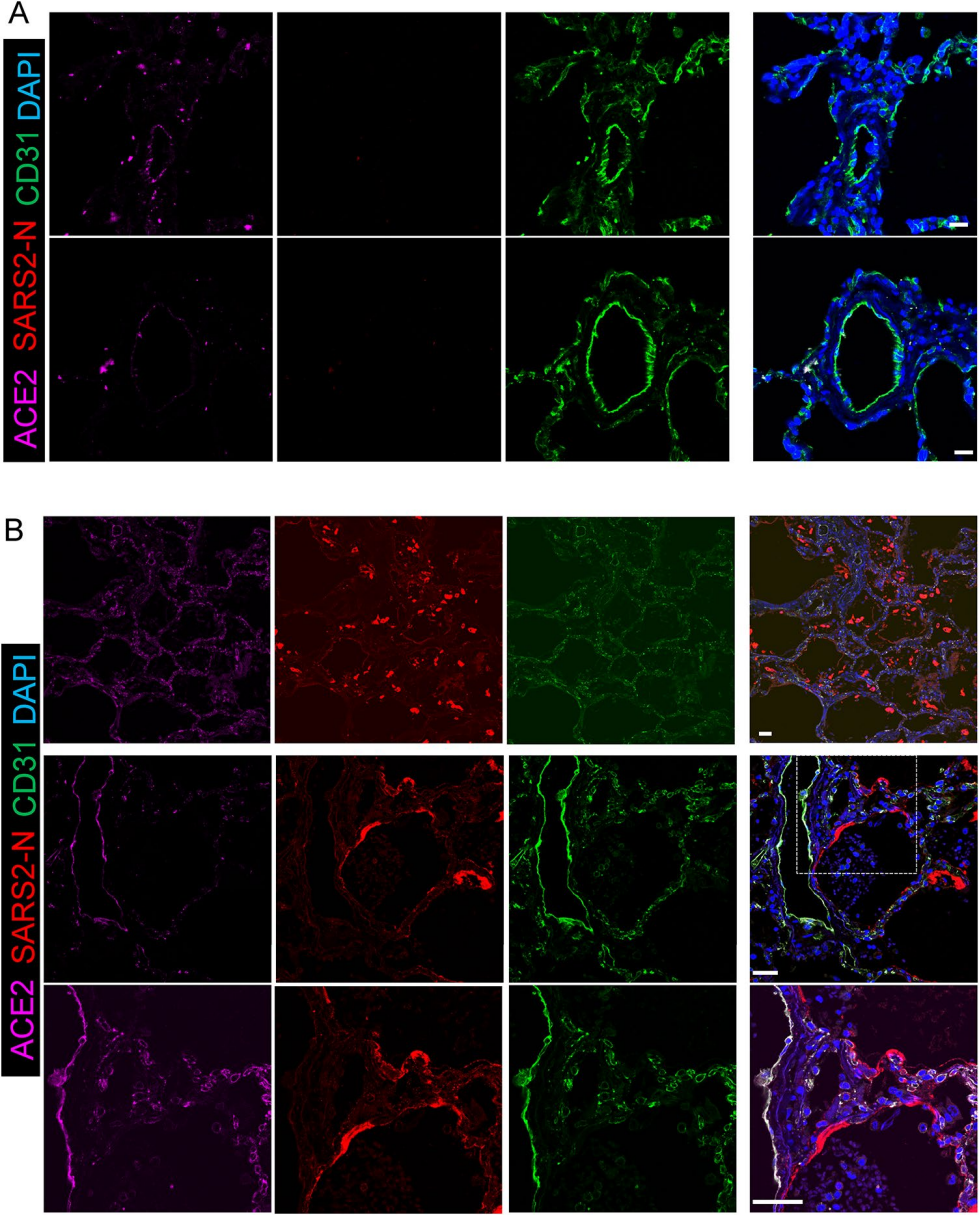
Immunofluorescence staining of ACE2, SARS-CoV-2 N, CD31 in non-COVID-19 and COVID-19 lungs. **A** Immunofluorescence of lung sections from human non-COVID-19 for ACE2 (magenta), SARS2-N (stain for SARS-CoV-2 nucleocapsid protein in red) and CD31 (stain for endothelium in green). DAPI serves as a nuclear DNA counterstain (blue). Bar = 20 µm. **B** SARS2-N shows Type I/II pneumocyte infection. In the top row, immunofluorescence of COVID-19 decedent lung sections from patient #3, stained for ACE2 (magenta), SARS2-N (stain for SARS-CoV-2 nucleocapsid protein in red) and CD31 (green). DAPI serves as a nuclear DNA counterstain (blue). Positive N staining shows acute phase of diffuse alveolar damage with sloughed alveolar Type I/II pneumocytes (200× magnification). In the middle row, immunofluorescence of COVID-19 decedent lung sections from patient #6 stained for ACE2, SARS2-N and CD31. Positive N staining shows type 1 cells with long processes along the air sacs (200× magnification). In the bottom row, zoomed insets showing the presence of viral infection in type 1 cells (630× magnification). Bar = 20 µm

**Fig. 2:**
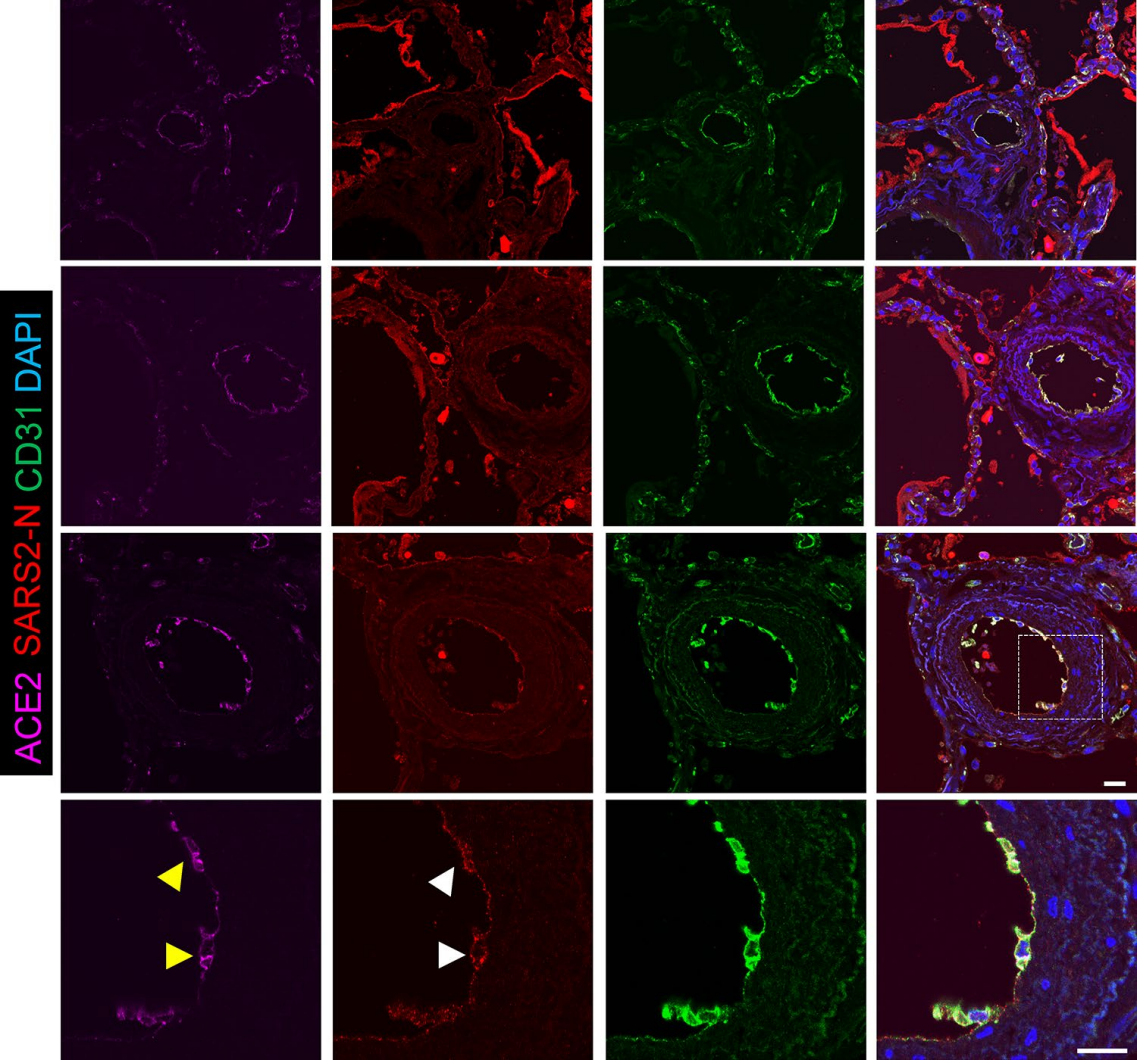
Endotheliitis is seen in tissue sections of COVID-19 lung endothelium. Immunofluorescence of COVID-19 decedent lung sections from patient #3, stained for ACE2 (magenta), SARS2-N (stain for SARS-CoV-2 nucleocapsid protein in red) and CD31(endothelium in green). Zoomed insets showing the presence of viral infection in these larger vessels (diameter > 100 µm) was consistently accompanied by signs of endothelial damage and vasculopathy, including disruption of the endothelial lining, sloughing of ECs into the vascular lumen, and apoptotic ECs with distorted nuclear shape (630×  magnification). Yellow arrowheads indicate positive ACE2 staining and white arrowheads indicate positive SARS2-N staining in the cytoplasm. ACE2, SARS2-N and CD31 are colocalized in an apoptotic EC. Bar = 20 µm

To evaluate the role of the pulmonary endothelium in this process, we applied super high-resolution microscopy and immunofluorescence (IF) staining to post-mortem lung samples from six adults (patient #1-#6) who died of complications of COVID-19. Consistent with the epidemiology of severe COVID-19, these individuals were older (ages 53–76), and all but one (patient #3) were hypertensive and/or diabetic and had histories of significant comorbidities (Table 1). Acute respiratory failure was a proximate cause of death in all six cases, and five of six cases had histopathologic evidence of diffuse alveolar damage at autopsy (Fig. 1, patient #3 as a representative). We assessed the vascular distribution of SARS-CoV-2 infection by IF staining of paraffin embedded lung samples with antibodies recognizing the SARS-CoV-2 N protein (anti-SARS-CoV-2 N) and the endothelial surface marker CD31 (anti-CD31) (Fig. 1). Though SARS-CoV-2 N staining was most predominantly distributed in Type I/II pneumocytes lining alveolar sacs and sloughed cellular debris in the airspaces (Fig. 1B inside air sacs with a bright red color), co-localization of anti-SARS-CoV-2 N and anti-CD31 antibodies consistent with endothelial SARS-CoV-2 infection was observed in samples from 4 of 6 subjects (Patient #1-#4, Fig. 1B; Suppl Figs. 1, 2, 3). The vascular distribution of endothelial viral infection was not uniform, observed mostly in larger (diameter > 100 µm) muscularized arteries (Fig. 2, 2nd and 3rd rows) and veins (Suppl Fig. 2, 3rd row) but absent in arterioles and vessels smaller than 50 µm in diameter (Fig. 2; Suppl Figs. 1, 2, 3, 4, 5, all 1st rows), including those in proximity to infection in adjacent pneumocytes. In these larger vessels, the presence of viral infection was consistently accompanied by signs of endothelial damage and vasculopathy, including disruption of the endothelial lining, sloughing of endothelial cells (ECs) into the vascular lumen, and apoptotic ECs with distorted nuclear shape (Fig. 2; Suppl Figs. 1, 2, 3, arrowheads). In the inner layer of blood vessels, we found the alignment of CD31 positive cells were discontinuous and most of them were sloughing off, thus the integrity of endothelial layers seen in patients #1-#4 was severely disrupted, whereas patients #5 and #6 demonstrated well preserved endothelial integrity and no obvious disruption. Additionally, no obvious vasculopathy was found in these two patients without evidence of SARS-CoV-2 endothelial infection (Suppl Figs. 4, 5), nor in the smaller vessels in patients (#1-4) with large-vessel endotheliitis, suggesting direct viral cytopathic effects as an inciting cause of damage. Though there were no obvious clinical differences between those with and without endotheliitis, histologic evidence of microvascular thrombosis was limited to those with endothelial damage in 3 of 4 subjects (Patient #1, 2, 3), including one subject (#3, Fig. 2) who was also found on post-mortem gross inspection to have a subsegmental pulmonary embolism. No evidence of thrombosis was observed in the two subjects (Patient #5, #6) who did not have SARS-CoV-2 vascular infection. Together, these findings suggested direct pulmonary endothelial infection with resultant endothelial damage as proximal events in the promotion of vascular thrombosis in COVID-19.

**Table 1:** Patient characteristics

	Patients with evidence of endothelial infection and damage				Patients without evidence of endothelial infection or damage	
	Patient 1	Patient 2	Patient 3	Patient 4	Patient 5	Patient 6
*Demographics*						
Age (Years)	66	57	68	76	58	53
Gender	F	M	F	M	M	M
Race	African American	Hispanic	White	White	White	White
Smoking status	Never smoker	Never smoker	Current smoker	Former pipe smoker	Former smoker	Former smoker
*Co-morbidities*						
BMI (kg/m2)	29.6	29.2	33.8	31.4	18.6	37.5
Hypertension	Yes	Yes	Yes	No	Yes	No
Diabetes	No	Yes	Yes	Yes	No	No
Other medical conditions						
Autoimmune/inflammatory	SLE, RA, ILD / PF, CKD, MGUS	None	Febrile neutropenia	No	No	No
Cardiopulmonary/vascular	CAD	Neurologic impairment	CAD, COPD	CAD, CHF, CKD, OSA	CF, CVA	OSA
*Medications*						
Immunosuppression	Prednisone, Tofacitinib	None	None	None	None	None
RAAS interacting drug	None	None	None	None	None	None
*COVID-19 course and management*						
Radiologic findings						
Ground glass (CT)	Bilateral	NA	Bilateral	NA	NA	NA
Consolidation (CT or CXR)	Yes	Bilateral (CXR)	Yes	No (CXR)	No (CXR)	Yes (CXR)
Mechanical ventilation	Yes	Yes	Yes	Yes	No	Yes
Ventilation mode	VC	VC	VC	AC	-	PC
PEEP(cmH2O)	14	22	10	16	-	15
FiO2	0.4	1	0.6	0.8	0.5	0.6
COVID-19 medication	Hydroxychloroquine, tocilizumab	No	No	Tocilizumab	No	Tocilizumab
Days hospitalized before death	7	1	1	7	2	5
Proximate causes of death	Respiratory failure and MSOF	Respiratory failure and MSOF	Respiratory failure	Respiratory failure and MSOF	Respiratory failure and SIRS	Respiratory failure and MSOF
*Pathologic findings*						
Lung weight, left/right (g)	630/910	1210/1220	580/580	1020/1560	430/510	1500/1850
Diffuse alveolar damage(DAD)	acute DAD(with scattered foci of organizing DAD) Interstitial lung disease with bronchiectasis	acute DAD (with scattered foci of organizing DAD)	acute DAD and prominent reactive pneumocyte hyperplasia	lung injury/DAD	Absent	acute DAD
Microvascular thrombi	Present	Present	Present	Absent	Absent	Absent

*BMI* body mass index, *SLE* systemic lupus erythematosus, *RA* rheumatoid arthritis, *ILD/PF* interstitial lung disease and pulmonary fibrosis, *CKD* chronic kidney disease, *MGUS* monoclonal gammopathy of unknown significance, *CAD* coronary artery disease, *COPD* chronic obstructive pulmonary disease, *CHF* congestive heart failure, *OSA* obstructive sleep apnea, *CF* cystic fibrosis, *CVA* cerebrovascular accident, *CT* computed tomography, *CXR* chest x-ray, *VC* volume control, *PC* pressure control, *PEEP* positive end-expiratory pressure, *FiO2* fraction inspired oxygen, *MSOF* multi-system organ failure, *SIRS* systemic inflammatory response syndrome

### No vascular infection was seen in K18-hACE2 transgenic mice

**Fig. 3:**
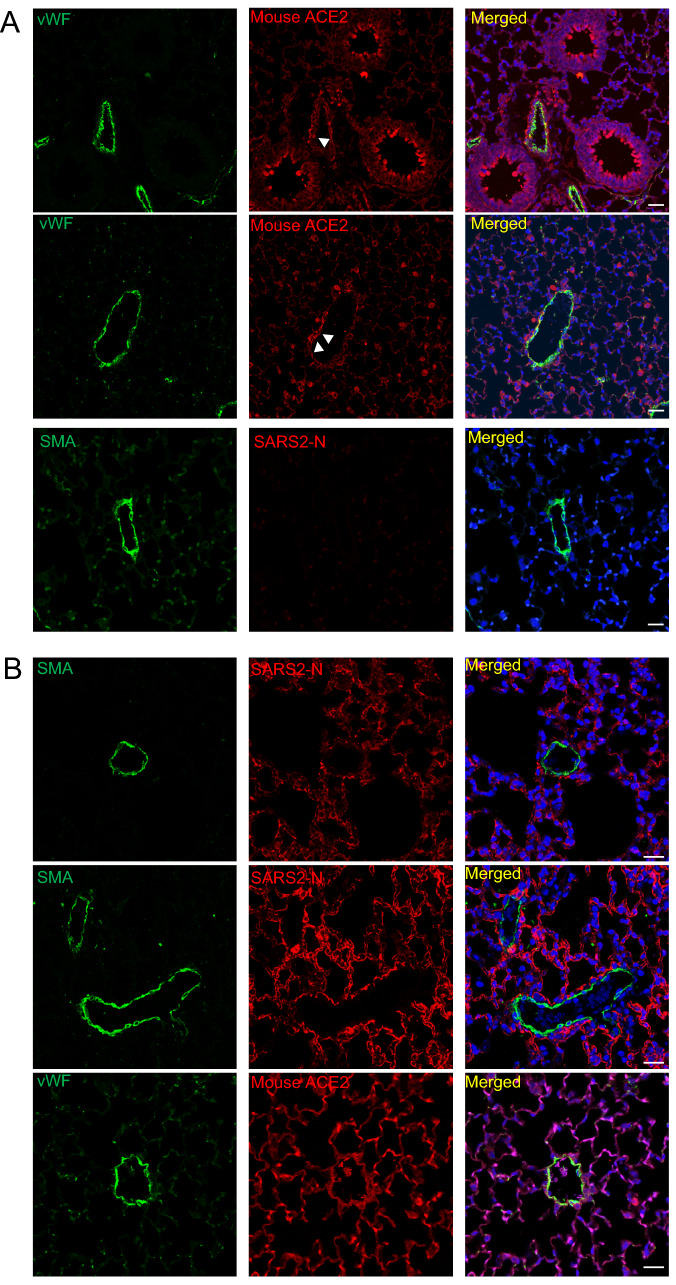
Immunofluorescence staining of mouse ACE2 and SARS2-N in tissue sections of K18-hACE2 transgenic mice. vWF (stain for endothelium in green), mouse ACE2 (red), SMA (stain for smooth muscle cell layer in green), SARS2-N (red) and DAPI serves as a nuclear DNA counterstain (blue). **A** Immunofluorescence of lung sections from uninfected K18-hACE2 transgenic mice. White arrowheads indicate co-localization of vWF and mouse ACE2 staining. Bar = 20 µm. **B** Immunofluorescence of lung sections from SARS-CoV-2-infected K18-hACE2 transgenic mice, 3.81 × 10^3^ TCID_50_ at 4 dpi. Bar = 20 µm

We further evaluated the vascular infection on K18-hACE2-transgenic mice, in which human ACE2 expression was driven only by the epithelial cell cytokeratin-18 (K18) promoter [8]. In control K18-hACE2 with mock infection, mouse ACE2 expression was predominantly positive in bronchial epithelial cells and type 2 pneumocytes and sparsely present on the endothelium (Fig. 3A, white arrowheads). The pattern of mouse ACE2 expression in K18-hACE2 was similar when compared to the wild-type C57/B6 strain (Suppl Fig. 6). After intranasal infection with 3.81 × 10^3^ TCID50 SARS-CoV-2 at 4 dpi, SARS-CoV-2 N staining was dominantly distributed in alveolar pneumocytes/air sacs but not present in any sizes of vessels (Fig. 3B). Thus, human ACE2 protein expression in the endothelia is a requirement for SARS-CoV-2 infection-induced vasculopathy.

### ACE2 expression was low/undetectable in non-COVID-19 pulmonary/systemic endothelial cells

**Fig. 4:**
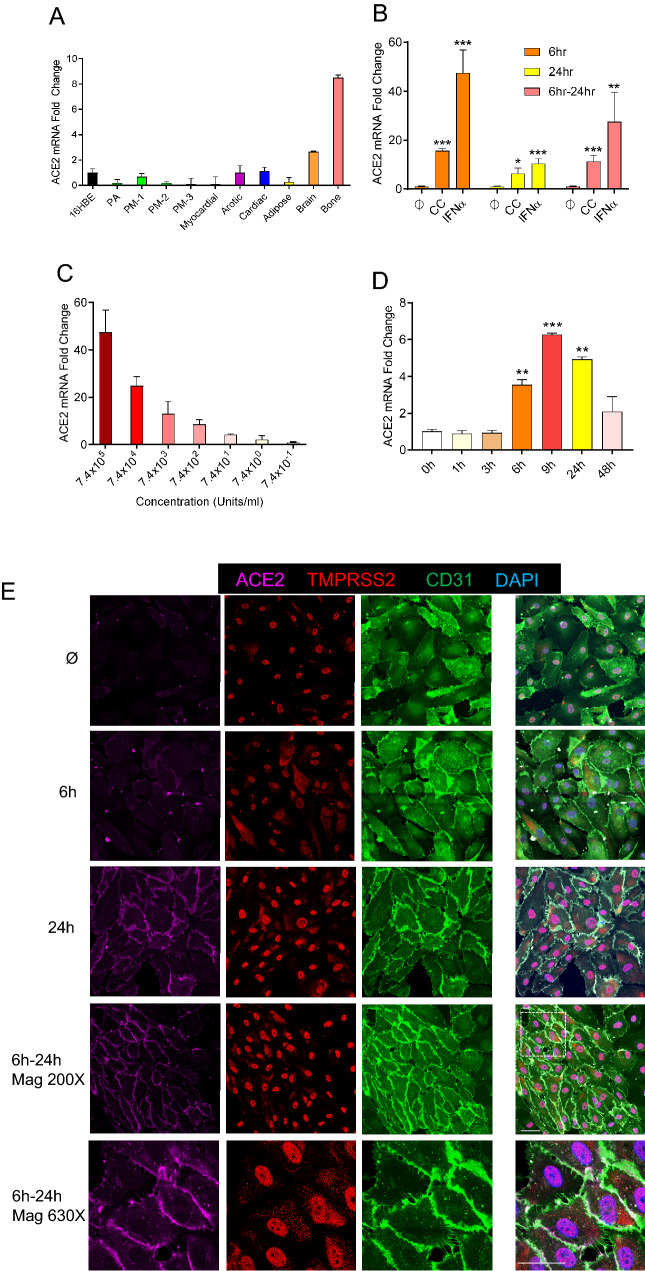
IFNα induces ACE2 in human primary endothelial cell cultures. **A** Relative mRNA expression of *ACE2* in different types of human endothelial cells, including the pulmonary arterial (PA), three pulmonary microvasculature (PM -1, -2, -3), myocardium (Myocardial), aorta (Aortic), cardiac microvascular (Cardiac), white adipose(Adipose), brain microvasculature (Brain) and bone (Bone). Endothelial expression is relative to that from the 16HBE human bronchial epithelial cell line (16HBE). Means ± SEM are from two technical replicates. **B** Relative mRNA expression of *ACE2* in human pulmonary arterial endothelial cells (PAECs) when incubated in cytokine-free media (ϕ); with a cytokine cocktail (CC—consisting of IFNα, IFNγ, TNFα, IL6 & CXCL10); or with IFNα alone for 6, 24 and 6–24 h (a total of 6 h simulation and then total RNA collect in 24 h). *ACE2* expression level in cytokine-free media were used as control. Means ± SEM derived from three biological replicates. *p < 0.05, **p < 0.01, ***p < 0.001 compared to untreated control, unpaired t test. **C** IFNα dose-dependent mRNA expression of *ACE2* in human PAEC. Fold-change of *ACE2* expression is relative to that in untreated cells. Means ± SEM are from two biological replicates. **D** Relative mRNA expression of *ACE2* in human PMVEC after IFNα incubation for 1, 3, 6, 9, 24 and 48 h, in a time-dependent manner. Means ± SEM are from two biological replicates. *p < 0.05, **p < 0.01, ***p < 0.001 compared to untreated control (0 h), unpaired t test. **E** Immunofluorescence for ACE2 (magenta) TMPRSS2 (red) and CD31 (green) in PAECs treated or not with IFNα (7.4 × 10^5^ units/mL) for 6, 24 and 6–24 h. Zoomed insets showing ACE2 expression on the cell membrane (630X magnification). Bar = 20 µm

The mechanism by which SARS-CoV-2 infects the endothelium was not obvious. SARS-CoV-2 cellular entry was dependent on the binding of its spike protein to ACE2 cell surface receptor, which facilitated transmembrane protease serine 2 (TMPRSS2) mediated spike protein cleavage and the induction viral-cellular membrane fusion. Though abundant on epithelial cells throughout the respiratory tract [9], ACE2 expression was more heterogeneous and weaker on the surface of ECs of the systemic circulation [10, 11]. Indeed, though staining with anti-ACE2 antibodies demonstrated widespread ACE2-positivity in pneumocytes in all of our post-mortem samples and in lung tissue samples from healthy controls, endothelium ACE2-positivity was more heterogeneous, and its mean fluorescence intensity was upregulated threefold in the SARS-CoV-2 infected samples (Fig. 2, Suppl Figs. 1, 2, 3, 4 5), but low/only rarely in the endothelium from non-COVID-19 samples (Fig. 1A). The observed low ACE2 expression in the endothelium of uninfected individuals was confirmed by publicly available single cell RNA-seq data, showing that *ACE2* mRNA was not detectable in the endothelium of most tissues studied, particularly the pulmonary endothelium, where no *ACE2* positive cells were identified among 7438 endothelial cells sampled (Suppl Tables 1, 2, 3). Similarly, screening for *ACE2* mRNA expression in primary ECs sampled from multiple tissues confirmed that under unstress conditions, though ECs sampled from systemic tissues showed a broad range of *ACE2* expression, *ACE2* expression was extremely low in either pulmonary arterial or pulmonary microvascular ECs (PAECs and PMVECs, respectively) but significantly higher in the primary brain and bone ECs (Fig. 4A).

### Interferon alpha induced pulmonary endothelial ACE2

**Fig. 5:**
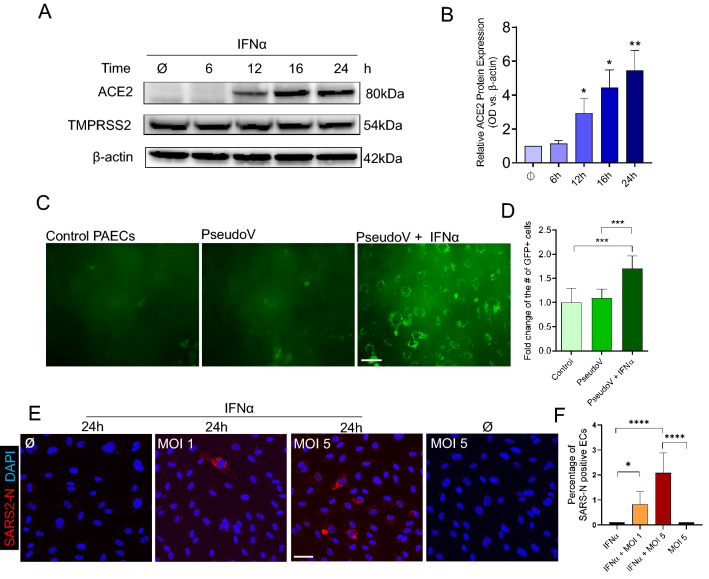
SARS-CoV-2 infection in human primary arterial endothelial cells. **A** Representative Western blot results of ACE2 and TMPRSS2 protein expression in PAECs treated with IFNα (7.4 × 10^5^ units/ml) for 6, 12, 16 and 24 h. 60ug total protein was loaded in each lane. β-actin serves as a loading control. **B** Optical Density (OD) quantification of ACE2 protein levels from A. Means ± SEM are from 4 biological replicates. *p < 0.05, **p < 0.01 compared to untreated, unpaired t test. **C** GFP expression in PAECs after d19/ R682Q modifications in Spike pseudoviral transduction (200×  magnification). Left: PAECs only; middle: without IFNα stimulation but transduced with pseudovirus; right: with IFNα stimulation and transduced with pseudovirus. Bar = 20 µm **D** Quantification of GFP positive cells vs. total number of cells from C. ***p < 0.001 compared to its control, unpaired *t* test. **E** Immunofluorescence images of viral nucleoprotein (SARS2-N, red) of PAECs infected with SARS-CoV-2 (MOI = 1 or 5) at 1 day post infection (200X magnification). Left: no IFNα stimulation but infected at MOI 5; middle left: IFNα 24 h but no viral infection; middle right: IFNα 24 h, infected at MOI 1; right: IFNα 24 h, infected at MOI 5. Bar = 20 µm. **F** Quantification of the number of positive SARS2-N cells (cytoplasmic red staining along with a nuclear DAPI staining) versus the total number of cells (the number of positive nuclear DAPI staining). Means ± SEM are calculated on six images from random fields with two biological replicates. *p < 0.001, ***p < 0.001, ****p < 0.0001 compared to its control, unpaired *t* test

It was worth noticing that viral burden positively correlated with proportions of venular endothelial cells and capillary aerocyte endothelial cells in single-cell atlases of post-mortem autopsy COVID-19 lungs [12]. Observing that ACE2 expression in the pulmonary vasculature was abundant in tissues from patients with fatal COVID-19 but none or moderately expressed in non-COVID-19 samples, we speculated that endothelial ACE2 expression was augmented in severe COVID-19 as a consequence of the host’s response to infection. Severe COVID-19 was frequently complicated by an uncontrolled inflammatory response—the so-called “cytokine storm”—including the excessive release of endotheliitis-associated chemokines, such as interleukin-6 (IL6), interferon alpha and gamma (IFNα and IFNγ), C-X-C Motif Chemokine Ligand 10 (CXCL10), and Tumor Necrosis Factor alpha (TNFα). High cytokine levels correlated with poor prognosis and high mortality in patients with COVID-19 [13, 14]. Given that the *ACE2* gene locus included multiple interferon response element sequences and IFNα can induce airway epithelial *ACE2* expression [15], we sought to evaluate whether one or more of these cytokines was responsible for upregulating ACE2 expression in severe COVID-19. Evaluating a range of conditions, we optimized our EC model system to ensure both PMVEC and PAEC cultures retained their fundamental physiological characteristics as a barrier and homeostasis gatekeeper, finding that 100% confluency was required for primary ECs to maintain their cubical cell shape and alignment, and form a tight junction monolayer (Suppl Fig. 7A, right panel). The addition of matrix-coating proteins, including fibronectin, rat tail or bovine collagen, and gelatin, did not enhance *ACE2* expression in these endothelial monolayers at the complete confluence when compared to plastic plates (Suppl Fig. 7B). Intriguingly, following the addition of a cytokine cocktail containing IL6, IFN (α or γ), CXCL10 and TNFα for 6 h, we observed sustained induction of endothelial ACE2 mRNA expression (Fig. 4B). Systematic permutation of the cytokine cocktail composition revealed that cocktails containing IFNα or IFNα alone significantly increased mRNA level of *ACE2* (Fig. 4B; Suppl Figs. 8, 9). Similar patterns were observed in both PMVECs and PAECs, confirming IFNα as both necessary and sufficient to induce endothelial *ACE2* mRNA expression. Dilution (Fig. 4C) and time-course (Fig. 4D) studies confirmed that IFNα was a potent, dose-dependent inducer of *ACE2* endothelial expression, with peak expression (45-fold increase from baseline) observed at 6 h, producing a sustained response which was observed at least 18 h after removal of the stimulus (by substituting for cytokine-free media at 6 h, noted as 6–24 h, Fig. 4B). By both IF staining of PAECs (Fig. 4E) and Western blot analysis of whole-cell lysates (Fig. 5A, B), we confirmed that endothelial ACE2 induction by IFNα resulted in ACE2 protein synthesis, with the greatest localization to the cytoplasmic membrane (Fig. 4E, 24 h and 6–24 h). Consistent with prior IF staining (Fig. 4E), there was no evidence of IFNα influencing the expression of TMPRSS2, the spike protein cleaving enzyme (Fig. 5A). Next, we decided to measure the biological function of endothelial cells upon IFNα stimulation. Compared with control, IFNα -stimulated PAECs demonstrated significantly increased leakage over 6 h. Finally, PAECs with IFNα resulted in shorter tube length, reduced branching points and less-organized tube networks compared with controls in Matrigel in 6 h (Suppl Fig. 10). Thus, IFNα adversely impacted on EC vessel formation, homeostasis, and barrier function.

### SARS-CoV-2 infected pulmonary endothelial cells

Having demonstrated the dependence of endothelial ACE2 expression on the presence of IFNα, we sought to determine its role in endothelial SARS-CoV-2 infection using our endothelial cell culture system and four pseudoviral constructs expressing the SARS-CoV-2 spike glycoprotein. We found that the spike protein pseudotyped viral constructs can be successfully transduced into ECs following 24 h stimulation with IFNα, and with more than 50% cells expressing GFP in each experiment (Fig. 5; Suppl Fig. 11). Importantly, our results were consistent with a previous report on between-strain differences in transduction efficiency [16]: viral constructs with the d19/R682Q modifications of the spike protein known to promote the most efficient viral entry demonstrated the greatest abundance GFP-expressing ECs in our studies (Fig. 5C, D). We next confirmed the ACE2 dependent viral infection in EC cultures with infectious SARS-CoV-2. Using IFNα-treated and untreated cells, EC cultures were infected with SARS-CoV-2 at a multiplicity of infection (MOI) of 1 or 5. By IF analysis using an antibody recognizing SARS-CoV-2 N, we found that whereas EC cells were not permissive to infection under basal conditions, ECs preconditioned with IFNα demonstrated a significant increase of N-positive cells by 2% (Fig. 5E, F), confirming that SARS-CoV-2 infection of the endothelial was dependent on induced ACE2 expression upon IFNα stimulation.

### Interferon alpha or beta induced ACE2 but not delta ACE2 in pulmonary endothelial cells

**Fig. 6:**
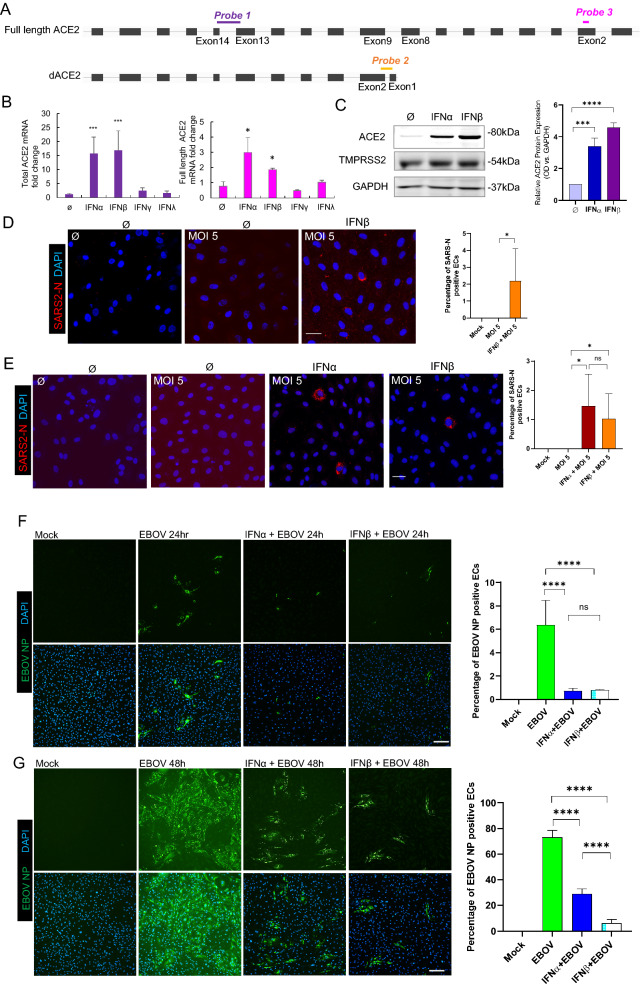
SARS-CoV-2 or Ebola virus infection of human primary PAECs after IFNα or β stimulation. **A** Schematic representation of ACE2 and delta ACE2(dACE2) transcripts and the position of the three PCR probes to generate wildtype ACE2, delta(d)ACE2 and full length ACE2 amplicons. **B** Expression of total ACE2 (left) and expression of full length ACE2 (right) in three PMECs after treated with IFNα, β, γ, λ for 6 h. **C** Representative Western blot results of ACE2 and TMPRSS2 protein expression in PAECs treated with IFNα or β for 24 h. 60 μg total protein was loaded in each lane. GAPDH serves as a loading control. Optical Density (OD) quantification of ACE2 protein levels vs GAPDH. Means ± SEM are from three biological replicates. **D** Immunofluorescence images of viral nucleoprotein (SARS2-N, red) of PAECs infected with SARS-CoV-2 (MOI = 5) at 1 day post infection (200X magnification). Left: Mock; middle: no IFNβ; right: IFNβ 24 h. Bar = 20 µm. **E** Immunofluorescence images of viral nucleoprotein (SARS2-N, red) of PAECs infected with recombinant SARS-CoV-2-mNeonGreen (MOI = 5) at 1 day post infection (200×  magnification). Left: Mock; middle left: no IFN; middle right: IFNα; right: IFNβ 24 h. Bar = 20 µm. **F** Immunofluorescence images of viral nucleoprotein (EBOV NP, green) of PAECs at 24 h post infection or at 48 h post infection (**G**). Cells were pretreated with IFNs for 24 h and infected with wildtype Ebola virus (EBOV) (MOI = 5) Left: Mock; middle left: no IFN; middle right: IFNα; right: IFNβ. Bar = 50 µm. Quantification (both **D**, **E**, **F** and **G**) of the number of positive SARS2-N or EBOV NP (cytoplasmic positive staining along with a nuclear DAPI staining) versus the total number of cells (the number of positive nuclear DAPI staining). Means ± SEM are calculated on six images from random fields with two technical replicates. *p < 0.05, ***p < 0.001 and ****p < 0.0001 compared to its control, unpaired t test

A previous study has described a transcriptionally independent truncated ACE2 isoform—delta ACE2 (dACE2)—incapable of binding the SARS-CoV-2 spike protein and proposed that only dACE2 was the sole interferon-inducible ACE2 isoform [17–19]. Given that our initial experiments (Fig. 4A–D, Supple Figs. 7, 8 9) employed primers that do not differentiate ACE2 isoforms, we repeated our measurements using two distally-positioned primer sets that distinguish between truncated dACE2(probe 2) and full length ACE2(probe 3) (Fig. 6A; Table 2). Ct values using for full length ACE2 (probe 3) were almost consistent to those obtained using the original assay from probe 1(total ACE2). In addition, the previously published probe 2 [17] did not detect any dACE2 mRNA expression (showed an undetermined Ct value). This demonstrates that IFNɑ induces transcription of full length ACE2. We further measured ACE2 using different types of IFNs. IFNβ, one of the Type I IFN family, upregulated mRNA of ACE2 up to 24-fold (0.075 µg/mL) or 39-fold (0.375 µg/mL) after 6 h simulation. Compared to other types of IFNs, only IFNα or β induced expression of total ACE2 or full-length ACE2 (Fig. 6B) whereas the no expression of dACE was observed (data not shown). The protein expression of ACE2 was also upregulated after 24 h IFNα or β stimulation by WB (Fig. 6C). Based on the prior condition (Fig. 5E), EC cultures were infected with SARS-CoV-2 at an MOI of 5 with or without IFNβ-treatment to determine the effect of IFNß treatment on SARS-CoV-2 susceptibility. By IF analysis using an antibody against SARS-CoV-2 N, we found that whereas EC cells were not permissive to infection under basal conditions, ECs preconditioned with IFNβ demonstrated a significant increase of N-positive cells (Fig. 6D). These results were further confirmed using recombinant SARS-CoV-2-mNeonGreen. After 48 h infection, ECs preconditioned with IFNα or β had increased of N-positive cells as shown by IF using an anti-N antibody (Fig. 6E). All these results implied that (at least in primary pulmonary ECs) IFNα and β but not other IFNs, induced the expression of functional ACE2 capable of the binding spike protein, proviral activity of IFNs. However, in contrast to highly susceptible cells in which SARS-CoV-2 N was expressed at high amounts and was homogenously distributed at late stages of infection [20], we observed a puncate distribution pattern of N in the IFN-treated ECs, suggesting that viral replication in theses cells was slightly impaired. A possible explanation for this could be a balance of pro- and antiviral activity of IFN in the infected ECs.

**Table 2:** Sequences and amplicons info of qPCR probe sequences for total ACE2, dACE2 and full length ACE2

Name	Forward	Reverse	Probe sequence	Amplicon size (bp)	Comment	References
Probe 1	GCCACTGCTCAACTACTTTG	GCTTATCCTCACTTTGATGCTTTG	ACTCCAGTCGGTACTCCATCCCA	124	Total ACE2	Commercial (IDT)
Probe 2	GGAAGCAGGCTGGGACAAA	AGCTGTCAGGAAGTCGTCCATT	AGGGAGGATCCTTATGTG	73	Only dACE2	Onabajo et al
Probe 3	ATAATGCTGGGGACAAATGG	TGTTCAACCGTTTGCTCTTG	TCCACACTTGCCCAAATGTA	346	Only full length	Self-design

To explore the effect of Type I IFN treatment of ECs on viruses that have been shown to be sensitive to IFN treatment and whose entry does not depend on the expression of ACE2, we infected IFN-treated ECs with Ebola virus (EBOV). EBOV is a member of the *Filoviridae* family and causes a severe hemorrhagic disease in humans with high fatality rates [21]. Entry of EBOV is mediated by several cell surface attachement factors and the intracellular receptor Niemann–Pick C1 (NPC1) with no dependence on ACE2 [22]. Treatment of cells with Type I IFNs renders cells resistant against EBOV infection [23] and has been shown to be beneficial for extended survival in infected nonhuman primates and Ebola virus disease patients [24, 25]. EC cultures were left untreated or treated with IFNα or β for the indicated times and infected with EBOV at an MOI of 5. By IF analysis using an anti-EBOV nucleoprotein (NP) antibody, we found that ECs preconditioned with IFNα or β for 24 h demonstrated a highly significant reduction of NP-positive cells at 24 and 48 h p.i. (Fig. 6F, G). Moreover, the EBOV infection rate in IFNβ-treated cells was fourfold decreased compared to IFNα-treated cells at 48 h p.i. (Fig. 6G). When cells were pretreated with IFN for 24 h prior to infection and and treated again at 24 h post infection, the reduction in EBOV infection rates was even more pronounced. As observed before, IFNβ treatment reduced the EBOV infection rate by fivefold compared to IFNα treatment (Suppl Fig. 12). These data show that IFNɑ and β retain their antiviral potential in our cell system independent of ACE2 regulation.

## Discussion

Our study suggested that endothelial damage likely occured secondary to infection in severe COVID-19. We first examined the ACE2 expression and viral infection of endothelium from six postmortem COVID-19 patient lungs using advanced microscopy. Compared with non-COVID-19 lungs, endothelial ACE2 expression was augmented in more severe COVID-19 infection as a consequence of the host’s response to infection. These stainings also showed, for the first time, that the vascular distribution of SARS-CoV-2 N protein was not uniform, observed mostly in larger (diameter > 100 µm) muscularized arteries and veins but absent in arterioles and vessels smaller than 50 µm in diameter. In these N-positive endothelia, endothelial damage and vasculopathy were consistently present, including disruption of the endothelial lining, sloughing of ECs into the vascular lumen, and apoptotic ECs with distorted nuclear shape (Fig. 2). Single cell RNA-seq data suggested that primary pulmonary endothelial cells were resistant to infection with SARS-CoV-2 due to the lack of ACE2 expression on the cell surface [26, 27]. Our results strongly support these prior findings and provide the first evidence that primary endothelial cells become only susceptible to SARS-CoV-2 after IFNα or β induced expression of functional ACE2 (not truncated form dACE2) (Figs. 5, 6). Our data also suggested that there was a balance between pro- and antiviral activities in the IFN-treated EC cultures. On one hand, IFNs facilitated ACE2-dependent SARS-CoV-2 entry, but on the other hand they restricted viral replication in the infected cells.

Many severe COVID-19 patients show signs of a cytokine storm, a hyperinflammatory response, which has been associated with causing the detrimental progression of COVID-19. IFNs are widely considered to be anti-viral agents, yet there is to date no substantial evidence of their clinical efficacy in treating COVID-19. Mechanistically, their contributions to host defense and maintenance of cellular homeostasis also remain unclear. The relationship between Type I(mainly α and β) and Type III (mainly λ) IFNs and COVID-19 severity remains controversial and complex. The pattern of IFN expression was measured at multiple sites, including nasopharyngeal swabs [28], bronchoalveolar lavage fluid [29], or peripheral blood [30], all of which revealed potent production of anti-viral IFN-stimulated genes (ISGs). Several studies showed that impaired IFN antiviral response in patients with severe and critical COVID-19, accompanied by high blood viral load and an excessive pro-inflammatory response [31, 32]. Nevertheless, some studies showed that dampened Type I IFNs response by autoantibodies predated SARS-CoV-2 infection and sharply increased infection prevalence in the elderly and/or severe cases [33].

In contrast to defective IFN responses, other studies have revealed caveats on the immunopathological role of IFNs and speculated on their temporal change from anti-viral spreading to tissue damage at the hyperinflammatory stage [34–36], thus heightened and prolonged production of IFNs was correlated with negative clinical outcomes. For instance, classic monocytes from severe COVID-19 exhibit a Type I IFN-driven signature that plays a pivotal role in exaggerating inflammation in severe-COVID-19 [30]. Type III IFN induces epithelial barrier damage, causing susceptibility to lethal bacterial superinfections [37]. In a multicenter cohort study, clinical administration of Type I IFN induces favorable clinical responses during the early stage of COVID-19, while later administration is associated with increased mortality [38]. A recent in-depth analyzed study highlighted the dynamic production of IFNs in the upper or lower respiratory tract of patients during COVID-19 disease progression. SARS-CoV-2 drove the production of Type III IFN in the upper airways as an IFN protection mechanism in younger and/or patients with mild disease. Moreover, critically ill patients expressed high levels of Type I IFN in the lower airways, but reduced induction of protective ISGs [39]. Furthermore, a NIH NIAID clinical trial had found that treatment of IFNβ did not improve outcomes for hospitalized patients with COVID-19 and some patients were even associated with more adverse events and worse outcomes. These findings provided us with novel insights on the opposing roles of IFNs and reconciled some contradictory findings on IFNs. We also speculated a preexisting vascular disease or genetic predisposition may also lead to these conflicting results. In our in vitro stimulation assay, the mRNA level of ACE2 was upregulated 15-fold by IFNα or 24-fold by IFNβ stimulation as short as 6 h. Intriguingly, after 6 h stimulation, IFNα was removed and replaced with regular culture media. The upregulated level of ACE2 mRNA was persistently upregulated for 23-fold for 24 h and 7-fold for 48 h. These observations had clinical implications, as endothelial ACE2 could be induced by Type I IFN in an immediate and persistent manner. More extensive and temporal studies should be carried out to further evaluate the molecular mechanisms on IFN inducible ACE2 signaling axis, including existing evidence on activated downstream pathways of PD-1, NFκb [40], 40].

Our study had some limitations. First, while endothelial cell infection and damage with SARS-CoV-2 was seen in the majority of patients analyzed, only six patients were included in our analysis and larger cohorts are needed, as findings may be contributed to other factors such as preexisting endothelial damage from comorbidities such as diabetes, hypertension, a predisposed genetic susceptibility to vasculopathy. Second, IFN levels in the six patients included were not obtained. As mentioned previously, longitudinal, prospective studies are therefore needed to characterize the temporal relationship between IFN levels, viral load, and endothelial ACE2 expression in COVID-19 infected patients. Third, while our study exclusively investigated the pulmonary vasculature after infection, additional experiments in other organ systems suffering from similar complications would provide more information on the complex relationship between COVID-19 infection of the endothelium. Finally, further in vivo and in vitro studies are needed to describe the downstream mechanism of how SARS-CoV-2 leads to endothelial cell apoptosis and denudation within the pulmonary vasculature. Recently, studies have suggested SARS-COV-2 infection may activate caspase-3 and 8 [42, 43], which triggers cell apoptosis in lung epithelial cells, however, studies on the endothelium are needed.

Though the viral induction of interferons and their downstream response genes are an early innate host response vital to containing most respiratory viral infections, our observations suggest that the release of IFNα or β in response to SARS-CoV-2 paradoxically facilitates the propagation of viral infection from respiratory epithelium to its surrounding vasculature, which in turn results in the endothelial damage that triggers the dysregulated coagulation and thrombotic complications that often drive poor patient outcomes. While the targeting of vascular IFNα or β signaling as a potential therapeutic strategy to prevent disease progression in patients with severe COVID-19 will require additional study, our findings raise a more pressing concern and reveal the mechanism regarding current treatment guidelines that propose the use of exogenous recombinant IFNα or β for the treatment of severe infection. Such recommendations should urgently be revisited.

## Materials and methods

### Study approval

The autopsy portion of this study was approved by the Institutional Review Board of Brigham and Women’s Hospital. The pseudoviral use of this study was approved by the Institutional Biosafety Committee of Boston Children’s Hospital.

### Clinical subjects

All autopsies were performed by one of the authors (RFP) at Brigham and Women’s Hospital on patients diagnosed with SARS-CoV-2 infection by nasopharyngeal swab RT-PCR either upon clinical presentation or post-mortem. The lungs were weighed, inflated with 10% neutral buffered formalin, sectioned and embedded in paraffin via standard procedures. Epidemiological, clinical, laboratory and radiological data were obtained from the electronic medical records.

### Immunohistochemistry

Postmortem lung tissues obtained from patients with COVID-19 were fixed in 10% neutral buffered formalin, paraffin embedded, sectioned at 5 μm, dewaxed, and rehydrated by standard procedures. Paraffin slides were placed into the vacuum at 60 degree Celsius for 20 min to achieve dewaxing. After, they were rehydrated by placing samples into Xylene and ethanol washes. Samples were then immediately placed into boiling 1 × Citrate Buffer (pH of 6.0) (C2488, Milipore/Sigma-Aldrich) for 10 min and following this allowed to sit in room air for 1 h. When at room temperature, samples were washed with 1X PBST(Tween20) and then blocked with donkey blocking serum for 1 h. Primary antibodies of interest, including sheep anti-CD31 (1:100; AF806, R&D systems), rabbit anti- SARS-N protein (1:500; 200-401-A50 Rockland), goat anti-ACE2 (1:50; AF933, R&D Systems), rabbit anti-vWF(1:300; Agilent A0082) were then applied at 4 degrees Celsius and allowed to incubate overnight. The next day, samples were washed five times for 10 min each in PBST and donkey blocking serum was applied at room temperature for 1 h. Secondary conjugate antibodies, donkey anti-sheep Alexa Fluor 488 (1:250; 713-545-003, Jackson Immunoresearch), donkey anti-rabbit Alex Fluor 555 (1:250; 711-165-152, Jackson Immunoresearch) and donkey anti-goat Alexa fluor 647 (1:250; 705-605-147, Jackson Immunoresearch) were applied for 6 h at room temperature. Slides were then washed five times with PBST again for 10 min each and mounted with DAPI (AF806, Vector Laboratories) to prepare for imaging. Following immunohistochemistry, the sections were imaged using Zeiss Confocal Z880 Airyscan microscopy.

K18-hACE2 transgenic mouse slides were provided by Dr. Hongpeng Jia (John Hopkins University). Paraffin slides were treated the same way as human slides. Primary antibodies of interest, including rabbit anti-vWF (1:200; A008202-5, Agilent), rabbit anti- SARS-N protein (1:500; 200-401-A50 Rockland), rat anti-mouse ACE2 (1:50; MAB3437, R&D Systems) and SMA-Alexa 647(1:50, sc-32251, Santa Cruz).

### Cell culture

Isolation of human ECs from adipose, bone, and myocardium tissues: Endothelial cells were isolated from normal human subcutaneous white adipose tissue, iliac crest corticocancellous bone tissue, and ventricular myocardial tissue. All these human samples were deidentified and discarded tissues obtained during clinical-indicated procedures in accordance with Boston Children’s Hospital Institutional Review Board-approved protocols. Tissues were minced and enzymatically (collagenase and dispase) digested for 2 h at 37 °C. Erythrocytes were lysed with RBC lysis buffer (New England Biolabs, Cat No. 420301). White adipose-, bone-, and myocardial-ECs were isolated by magnetic activated cell sorting (MACS) using CD31-coated magnetic beads (Dynabeads, Invitrogen, Cat No. 11155D). Isolated ECs were cultured on 1% gelatin-coated plates using EC medium: EGM-2 (except for hydrocortisone; PromoCell, Cat No. C22111) supplemented with 10% GenClone FBS (Genesee, Cat No. 25-514) and 1 × glutamine-penicillin–streptomycin (GPS, ThermoFisher, Cat No. 10378106). Three human pulmonary microvascular endothelial cells are purchased from PromoCell(C-12282). Human pulmonary artery endothelial cells (3100), human Aortic Endothelial cells (6100), human cardiac microvascular endothelial cells (6000), human brain microvascular endothelial cells (1000) are purchased from ScienCell and maintained in Endothelial Cell Medium (ECM, 1001, ScienCell), supplemented with 5% fetal bovine serum (0025, ScienCell), 1% penicillin/streptomycin (P/S, 0503, ScienCell), and Endothelial Cell Growth Supplement (ECGS, 1052, ScienCell) and incubated at 37 °C in an incubator with an atmosphere of 5% CO2/95% air. Cells were digested with 0.25% trypsin EDTA (MT25053CI, Corning) and passaged when reaching 90% confluency. All experiments were performed with ECs before passage six. Gelatin (1:2, Sigma G1393), Type 1 bovine collagen (1:60, Advanced BioMatrix, Cat #5005), Cultrex Bovine Collagen I (1:100, R&D 3442-050-01), fibronectin (1:100, Sigma F1141) are diluted in 1X PBS and used for coating at least 1 h at room temperature.

### Cytokine stimulation

The day before, coat the culture plates with 1% gelatin for 1 h and seed a substantial number of cells on the coated culture plates. The next day, EC must form a monolayer to proceed with the stimulation. The concentration of cytokine cocktail are made of recombinant human IFN-alpha A (alpha 2a) (7.4 × 10^5^ units/mL, R&D cat# 11100-1) IFNγ (0.5ug/mL, Peprotech #300-02), TNFa(0.1 μg/mL, R&D 210-TA-020), IL6(0.1 μg/mL, Peprotech #200-06), CXCL10 (0.1 μg/mL, Peprotech #300–12). After incubation cytokines with a determined time point, total RNA will be collected and proceed with cDNA reverse transcription.

### Pseudoviral tranduction

The SARS-CoV-2 S protein cDNA was used to pseudotyped human immunodeficiency virus (HIV) expressing an enhanced green fluorescence by using previously described methods [16]. A vesicular stomatitis virus G (VSV G) protein pseudotyped HIV expressing eGFP was used as positive control for viral transduction. The day before viral transduction, the culture plates (ibidi 3 well insert Cat# 80366) were coated with 1% gelatin for 1 h and seed 12,000 cells on the coated culture plates. The next day, EC must form a monolayer to proceed with the IFNα stimulation. IFNα (1:120) diluted in full EC media and incubated on cells for 24 h. Wash cells with 1XPBS three times and dilute any of three pseudovirus 1:5 ratio in serum free and ECGS free medium. Incubate pseudovirus solution on cells for 2 h. Remove the viral solution, wash 3X with 1X PBS, and change back to full EC medium. GFP signal is acquired after 24 h using Zeiss Axio Observer Z1 Inverted Epifluorescence Microscope. Live cellular DAPI staining (Invitrogen R37605) is added to the medium to counterstain the nuclei.

### Virus propagation and titration

SARS-CoV-2 (isolate USA_WA1/2020) was kindly provided by CDC’s Principal Investigator Natalie Thornburg and the World Reference Center for Emerging Viruses and Arboviruses (WRCEVA). Recombinant SARS-CoV-2 expressing mNeonGreen (SARS-CoV-2-mNG) was kindly provided by Pey-Yong Shi, University of Texas Medical Branch, Galveston and the World Reference Center for Emerging Viruses and Arboviruses [44]. This virus is based on SARS-CoV-2 isolate SARS-CoV-2 isolate USA_WA1/2020. EBOV (isolate Mayinga) was kindly provided by Heinz Feldmann, NIH NIAID Rocky Mountain laboratories. All virus stocks were propagated in Vero E6 cells (ATCC CRL-1586) cultured in Dulbecco’s modified Eagle’s medium (DMEM) supplemented with 2% fetal calf serum (FCS), penicillin (50 U/ml), and streptomycin (50 mg/mL). Viral stocks were purified by ultracentrifugation through a 20% sucrose cushion at 80,000× *g* for 2 h at 4 °C as described before [20]. Viral titers were determined in Vero E6 cells by tissue culture infectious dose 50 (TCID50) assay using the Spearman and Kärber algorithm. All work with SARS-CoV-2 and EBOV was performed in the biosafety level 4 (BSL4) facility of the National Emerging Infectious Diseases Laboratories at Boston University, Boston, MA following approved SOPs.

### Viral infection of slides

PAECs were seeded at a density of 5.2 × 10^4^ cells per well in 8-well ibidi slides (Cat# 80,826) in full ECM (ECM, 1001, ScienCell). For infection, cell supernatants were removed and replaced with 120 µL of ECM without FBS or growth supplement containing SARS-CoV-2, SARS-CoV-2-mNG or EBOV. Cells were infected at an MOI of 1 or 5 for 2 h. Infection medium without virus was used as mock control. After a 2 h incubation period at 37 °C in 5% CO_2_, inoculum was removed, and cells were washed once with PBS. 300 µl of full ECM were added per well, and cells were incubated at 37 °C, 5% CO_2_ until fixation. For additional treatment with IFNs, IFNɑ or ß was added into corresponding wells at 24 h post infection. For fixation, cell supernatants were removed, and cells were washed once with PBS and fixed in 10% formalin for at least 6 h and removed from the BSL-4 laboratory in accordance with approved SOPs. Briefly, EBOV staining was performed using anti-EBOV-NP (IBT Bioservices, 1:200 overnight) and followed with goat-anti-rabbit-AF488 (Invitrogen 1:200, 1 h) and the mounting media containing DAPI.

### RNA extraction and qPCR

RNA of multiple primary human endothelial cells was extracted using RNeasy Mini Kit (74,106, Qiagen), and reverse transcribed with High-Capacity cDNA Reverse Transcription Kit (4,374,966, Applied Biosystems). Quantitative RT-PCR was performed on QuantSudioTM 7 Flex Real-time System (Applied Biosystems) with TaqMan probes predesigned by Integrated DNA technologies. Relative expression level of ACE2 was calculated based on the standard 2 − ΔΔCT method using GAPDH as a reference gene. Gene expression comparisons were performed using unpaired t-test.

### Western blot

Total protein (60 μg) extracted from human pulmonary artery endothelial cells was separated by 4–12% SDS-PAGE (NP0321, Invitrogen), transferred to a PVDF membrane, and immunoblotted with goat polyclonal ACE2 antibody (AF933, R&D), rabbit anti-TMPRSS2 antibody (ab92323, Abcam) or mouse anti-beta Actin antibody (ab49900, Abcam) as a loading control. Anti-goat, anti-rabbit and anti-mouse HRP antibodies were purchased from Abcam. Protein bands were detected using the LAS-4000 luminescent imaging system (Fujifilm Life Science) and quantified with Image J software. Protein expression comparisons were performed using unpaired t-test.

### Fluorescein isothiocyanate (FITC)—dextran permeability assay

0.4 µm-pore inserts in a 24-well plate (Costar #38024) were purchased. Before the experiment, inserts were coated with 1% gelatin in room temperature for 1 h. 2.5 × 10^4^ PAECs were plated onto the collagen-coated inserts in a 37 °C/5% CO2 tissue culture incubator. After overnight incubation, IFNα (500U/μL) was added on apical side. After 24-h IFNα treatment, a solution of 1 μg/mL FITC-Dextran (MW: 70 kDa; Sigma) in 100µL full ECM was added in the inserts and 600µL full ECM in the lower chamber. Fluorescence intensity was detected every hour up to 6 h by using the Microplate Reader (Tecan Spark multimode microplate reader; Switzerland) with excitation and emission wavelengths of 492 and 520 nm, respectively.

### Matrigel angiogenesis assay

Matrigel (356,231, Corning) was thawed on ice overnight, loaded 10 µL each well on µ-Slide Angiogenesis glass bottom plate (Ibidi product #81507, Munich, Germany), and incubated for 1 h at 37 °C for polymerization. 3 × 10^3^ PAEC alone or 3 × 10^3^ PAEC mixed IFNα in 50 µL ECM were dispersed on each Matrigel-coated well. Images were captured every hour up to 4 h by using a Leica Inverted microscope. Total tube lengths, total branching points and number of loops were quantified using AngioTool and ImageJ.

### Statistics

Statistical analyses were performed using GraphPad Prism 7. Unpaired Student’s t-test was used for two-group comparisons. All stimulated groups are compared to the relevant control group. More details are described in the figure legends.

## Supplementary Information

Below is the link to the electronic supplementary material.

Supplementary file1 (DOCX 2650 kb)
