# Supplementary material for: Interferon-alpha or -beta facilitates SARS-CoV-2 pulmonary vascular infection by inducing ACE2
Source: Angiogenesis. 2021 Oct 29;25(2):225–40. doi: 10.1007/s10456-021-09823-4 (PMC8554520; doi:10.1007/s10456-021-09823-4)
Supplement: Supplementary file 1 — Supplementary file1 (DOCX 2650 kb) [file 10456_2021_9823_MOESM1_ESM.docx]

Supplementary Figure 1. Immunofluorescence of COVID-19 decedent lung sections from patient #1, stained for ACE2(magenta), SARS2-N(red) and CD31(green). Yellow arrowheads indicate positive ACE2 staining and white arrowheads indicate positive SARS2-N staining in the cytoplasm. DAPI serves as a nuclear DNA counterstain (blue). Bar=20 µm.

Supplementary Figure 2. Immunofluorescence of COVID-19 decedent lung sections from patient #2, stained for ACE2(magenta), SARS2-N(red) and CD31(green). The top two rows are arteries, the third row is a vein. Yellow arrowheads indicate positive ACE2 staining and white arrowheads indicate positive SARS2-N staining in the cytoplasm. DAPI serves as a nuclear DNA counterstain (blue). Bar=20 µm.

Supplementary Figure 3. Immunofluorescence of COVID-19 decedent lung sections from patient #4, stained for ACE2(magenta), SARS2-N(red) and CD31(green). The top two rows are arteries, the third row is a vein. Yellow arrowheads indicate positive ACE2 staining and white arrowheads indicate positive SARS2-N staining in the cytoplasm. DAPI serves as a nuclear DNA counterstain (blue). Bar=20 µm.

Supplementary Figure 4. Immunofluorescence of COVID-19 decedent lung sections from patient #5, stained for ACE2(magenta), SARS2-N(red) and CD31(green). No disrupted endothelial structure is observed. DAPI serves as a nuclear DNA counterstain (blue). Bar=20 µm.

Supplementary Figure 5. Immunofluorescence of COVID-19 decedent lung sections from patient #6, stained for ACE2(magenta), SARS2-N(red) and CD31(green). No disrupted endothelial structure is observed. DAPI serves as a nuclear DNA counterstain (blue). Bar=20 µm.

Supplementary Figure 6. Immunofluorescence staining of mouse ACE2 and SARS2-N in tissue sections of wildtype C57/B6 mice. vWF (stain for endothelium in green) and mouse ACE2 (red), and DAPI serves as a nuclear DNA counterstain (blue). White arrowheads indicate co-localization of vWF and ACE2 staining. Bar=20 µm


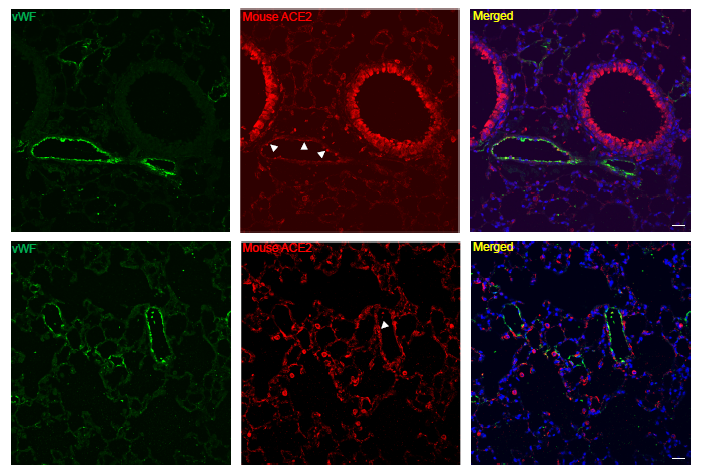
Supplementary Figure 7. mRNA expression of *ACE2* in pulmonary endothelial cell cultures. (A) PMVECs in a non-confluent vs a confluent monolayer culture condition (100X magnification). Bar = 50µm. (B) Expression of *ACE2* in PMECs with various coating conditions (collagen I, Fibronectin, Gelatin) compared to plastic plates after treated with IFNα (7.4x10^5^ units/mL) for 6 h. Means±SEM are from 2-3 biological replicates. Relative gene expression by RT-qPCR, calculated using the comparative Ct method with *GAPDH* as the control gene (2^-ΔΔCt^ method). Means±SEM are from three biological repeats. * p<0.05, ** p<0.01, *** p<0.001 compared to its control, unpaired t test.


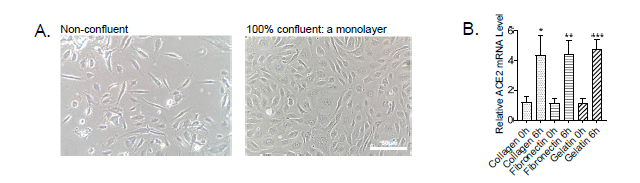
Supplementary Figure 8. IFNα is identified as the individual cytokine that induces *ACE2* expression from different permutations of cytokine combinations in human PMECs. Relative mRNA expression of *ACE2* when incubated for 6 hours with one cytokine (A), two-cytokine combinations (B), three-cytokine combinations (C), and four-cytokine combinations (D). The concentrations of each cytokine are as follow: IFNα 7.4x10^5^ units/mL, IFNγ 0.5 µg/mL, TNFα 0.1 µg/mL, IL6 0.1 µg/mL, CXCL10 0.1 µg/mL. The untreated group is applied as control. Means±SEM are from two technical replicates. Solid circle indicated application of relevant cytokine, and empty circle indicated no cytokine added.

Supplementary Figure 9. IFNα is identified as the individual cytokine that induces *ACE2* expression from different permutations of cytokine combinations in human PAECs. Relative mRNA expression of *ACE2* when incubated for 6h with one cytokine (A), two-cytokine combinations (B), three-cytokine combinations (C), and four-cytokine combinations (D). The concentrations of each cytokine are as follow: IFNα 7.4x10^5^ units/mL, IFNγ 0.5µg/mL, TNFα 0.1µg/mL, IL6 0.1µg/mL, CXCL10 0.1µg/mL. The untreated group was applied as control. Means±SEM are from 2 technical replicates. Solid circle indicated application of relevant cytokine, and empty circle indicated no cytokine added.

Supplementary Figure 10. IFNα adversely impacts on EC permeability, tube formation and barrier function. (A), PAECs show increased Fluorescein Isothiocyanate (FITC) leakage in the lower wells after 2hr IFNα stimulation and continuous increased absorbance (indication of leakage) till 6hr by Dextran Permeability Assay (B), PAEC tube formation with or without IFNα by Matrigel assays. Total tube length (left), total branching points (middle), and number of loops (right) were quantified. Means±SEM are calculated on six images from random fields with three biological replicates. * p<0.05, ** p<0.01 and *** p<0.001 compared to its control, unpaired t test, Bar =100 µm.


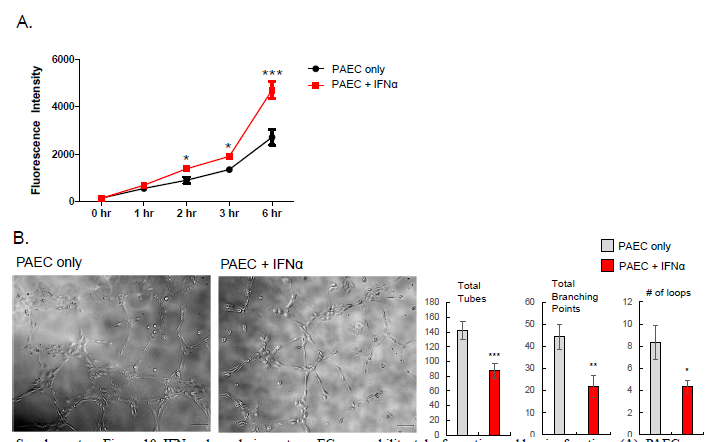
Supplementary Figure 11. PAECs are transduced with pseudotyped viruses and expressing GFP. Top row: SARS-CoV-2 delta19 Spike pseudotyped HIVexpressing eGFP; bottom row: vesicular stomatitis virus G (VSV G) protein pseudotyped HIV expressing eGFP. Left column: neither IFNα nor pseudoviral added; middle: no IFNα but transduced with pseudovirus; right: with IFNα 24hr treatment and transduced with pseudovirus. The graphs on the right represent the quantification of GFP+ cells versus total number of cells for each pseudovirus. GFP (green) and DAPI serves as a nuclear DNA counterstain (blue). 400X magnification.


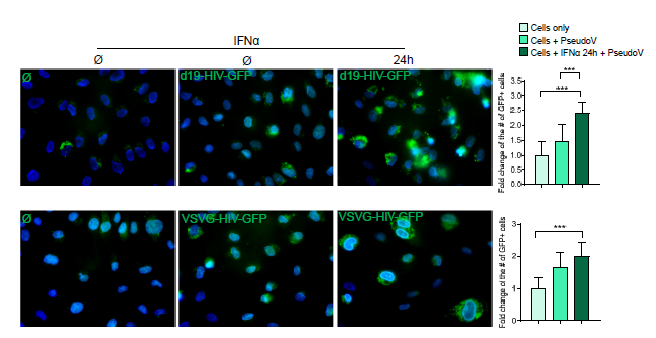
Supplementary Figure 12. Immunofluorescence images of viral nucleoprotein (EBOV NP, green) of PAECs at 48hr post infection. Cells were pretreated with IFNs for 24hr and infected with Ebola virus (MOI=5). At 24hr post infection (hpi), the cells were treated again with IFNs. Cells were fixed at 48 hpi. Left: Mock; middle left: no IFN, EBOV-infected; middle right: IFNα added at 24 hpi; right: IFNβ added at 24 hpi. Bar =50 µm. Quantification of the number of positive EBOV NP (cytoplasmic positive staining along with nuclear DAPI staining) versus the total number of cells (the number of positive nuclear DAPI staining). Means±SEM are calculated on six images from random fields with two technical replicates. **** p<0.0001 compared to its control, unpaired t test.


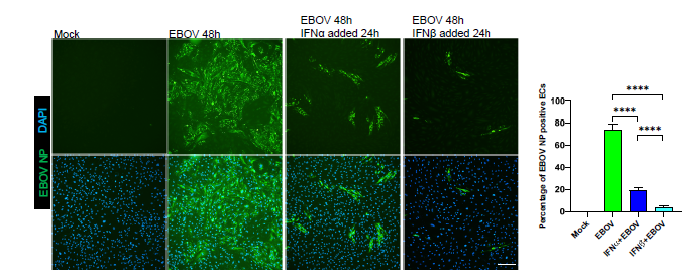


Supplementary Table 1. ACE2 Endothelial Expression in the lung in publicly-available scRNA-Seq Datasets

| **Database** | **EC type** | **Cell count**  **(ACE2 positive ECs / total ECs)** | **Mean ACE2 expression** | **Reference** |
| --- | --- | --- | --- | --- |
| 1. Human Cell Atlas | Unspecified | 0 / 744 |  | [45] |
|  | Venous | 0 / 254 |  | N/A |
|  | Unspecified | 0 / 592 |  | N/A |
|  | Unspecified | 0 / 733 |  | N/A |
|  | Lymphatic  Vascular | 0/461  0/1265 |  | N/A  N/A |
|  | Lymphatic | 0/222 |  | [46] |
|  | Lymphatic | 0/349 |  | [47] |
|  | Lymphatic | 0/395 |  | [48] |
|  | Lymphatic | 0/336 |  | [49] |
| 2. IPF Cell Atlas | Arterial, capillary, venous | N/A | 0.005-0.035 | [46-48, 50, 51] |
| 3. CellGene | Artery  Lymphatic | 0/92  0/44 |  |  |
|  | Artery  Lymphatic | 0/1484  0/467 |  |  |
| 4. Lung Endothelial Cell Atlas | Aerocyte  Arterial  General capillary  Venous | N/A | Human ACE2 gene is not included | [46, 48, 52-54] |
| 5. LungMap | Vascular  Adult (24 y.o.)  Infant (1 day) |  | 2.79  0.34 | N/A |
| Review and interpretation of five publically available single-cell RNA-sequencing datasets on lung tissue. Databases searched for ACE2 expression on endothelial cells and summarized above. Mean ACE2 expression without units. Hyperlinks to data provided in Supplemental Table 3. | | | | |

Supplementary Table 2: The hyperlinks of scRNA Public Databases for ACE2 Endothelial Expression in the Lung

| Database | Link |
| --- | --- |
| Human Cell Atlas | <https://singlecell.broadinstitute.org/single_cell/study/SCP867/hca-lungmap-covid-19-barbry-lung?scpbr=hca-covid-19-integrated-analysis> |
|  | https://singlecell.broadinstitute.org/single_cell/study/SCP873/hca-lungmap-covid-19-groningen-nawijn-2019-vieira-braga?scpbr=hca-covid-19-integrated-analysis |
|  | https://singlecell.broadinstitute.org/single_cell/study/SCP875/hca-lungmap-covid-19-internal-nonsmokers-lung?scpbr=hca-covid-19-integrated-analysis |
|  | https://singlecell.broadinstitute.org/single_cell/study/SCP876/hca-lungmap-covid-19-smokers-lung?scpbr=hca-covid-19-integrated-analysis |
|  | https://singlecell.broadinstitute.org/single_cell/study/SCP878/hca-lungmap-covid-19-lung-cross-tissue?scpbr=hca-covid-19-integrated-analysis |
|  | https://singlecell.broadinstitute.org/single_cell/study/SCP879/hca-lungmap-covid-19-misharin-new?scpbr=hca-covid-19-integrated-analysis |
|  | https://singlecell.broadinstitute.org/single_cell/study/SCP881/hca-lungmap-covid-19-northwestern-misharin-2018-reyfman?scpbr=hca-covid-19-integrated-analysis |
|  | https://singlecell.broadinstitute.org/single_cell/study/SCP886/hca-lungmap-covid-19-pittsburgh-lafyatis-2019-morse?scpbr=hca-covid-19-integrated-analysis |
|  | https://singlecell.broadinstitute.org/single_cell/study/SCP897/hca-lungmap-covid-19-vanderbilt-kropski-biorxiv-habermann?scpbr=hca-covid-19-integrated-analysis |
|  | https://singlecell.broadinstitute.org/single_cell/study/SCP900/hca-lungmap-covid-19-sanger-meyer-2019-madissoon?scpbr=hca-covid-19-integrated-analysis |
| IPF Cell Atlas | http://www.ipfcellatlas.com/ |
| CellGene | https://cellxgene.cziscience.com/e/krasnow_lab_human_lung_cell_atlas_smartseq2-2-remixed.cxg/ |
|  | https://cellxgene.cziscience.com/e/krasnow_lab_human_lung_cell_atlas_10x-1-remixed.cxg/ |
| Lung Endothelial Cell Atlas | http://www.lungendothelialcellatlas.com |
| LungMap | https://lungmap.net/breath-entity-page/?entityType=gene&entityId=Gene_ID_59272&entityLabel=ACE2 |

Supplementary Table 3. ACE2 Endothelial Expression in Other Organ Systems: Review of scRNA Public Databases

| Database | Tissue | ACE2 expression on ECs / Total ECs | Link | Reference |
| --- | --- | --- | --- | --- |
| Human Cell Atlas^#4^ | Cardiac | Lymphatic EC (0/660) | https://singlecell.broadinstitute.org/single_cell/study/SCP846/myocyte-specific-upregulation-of-ace2-expression-in-cardiovascular-disease-implications-for-sars-cov-2-mediated-myocarditis?scpbr=hca-covid-19-integrated-analysis | [55] |
|  | Adipose | Endothelial-1 (0/207)  ACKR1+ Endothelial-2 (0/863) | https://singlecell.broadinstitute.org/single_cell/study/SCP865/hca-lungmap-covid-19-adipose?scpbr=hca-covid-19-integrated-analysis | N/A |
|  | Kidney | Endothelium Vascular (0/429)  Endothelium lymphatic (1/160) | https://singlecell.broadinstitute.org/single_cell/study/SCP868/hca-lungmap-covid-19-kidney?scpbr=hca-covid-19-integrated-analysis | N/A |
|  | Enteric Nervous System | N/A | https://singlecell.broadinstitute.org/single_cell/study/SCP869/hca-lungmap-covid-19-ens?scpbr=hca-covid-19-integrated-analysis | N/A |
|  | Esophagus  Mucosa | Endothelium Vascular (0/889)  Endothelium lymphatic (0/888) | https://singlecell.broadinstitute.org/single_cell/study/SCP870/hca-lungmap-covid-19-esophagus-mucosa-cross-tissue?scpbr=hca-covid-19-integrated-analysis | N/A |
|  | Esophagus Muscle | Endothelium Lymphatic (0/844)  Endothelium Vascular (0/844) | https://singlecell.broadinstitute.org/single_cell/study/SCP871/hca-lungmap-covid-19-esophagus-muscularis-cross-tissue?scpbr=hca-covid-19-integrated-analysis | N/A |
|  | Heart cross tissue | Endothelium vascular (0/1610)  EC lymphatic (0/657) | https://singlecell.broadinstitute.org/single_cell/study/SCP871/hca-lungmap-covid-19-esophagus-muscularis-cross-tissue?scpbr=hca-covid-19-integrated-analysis | N/A |
|  | Liver | Periportal endothelial (1/819) | https://singlecell.broadinstitute.org/single_cell/study/SCP877/hca-lungmap-covid-19-liver?scpbr=hca-covid-19-integrated-analysis | N/A |
|  | Nasal polyps | Endothelial (1/545) | https://singlecell.broadinstitute.org/single_cell/study/SCP880/hca-lungmap-covid-19-nasal-polyps?scpbr=hca-covid-19-integrated-analysis | [56] |
|  | Pancreas | Endothelial (0/23) | https://singlecell.broadinstitute.org/single_cell/study/SCP883/hca-lungmap-covid-19-pancreas?scpbr=hca-covid-19-integrated-analysis | [57] |
|  | Pancreatic ductal adenocarcinoma Naïve | Endothelial (0/869)  Nascent endothelial (5/344) | https://singlecell.broadinstitute.org/single_cell/study/SCP884/hca-lungmap-covid-19-pdac-naive?scpbr=hca-covid-19-integrated-analysis | N/A |
|  | Pancreatic ductal adenocarcinoma treated | Endothelial (3/884) | https://singlecell.broadinstitute.org/single_cell/study/SCP885/hca-lungmap-covid-19-pdac-treated?scpbr=hca-covid-19-integrated-analysis | N/A |
|  | Prostate | Endothelial vascular (0/1216)  Endothelial Lymphatic (0/273) | https://singlecell.broadinstitute.org/single_cell/study/SCP887/hca-lungmap-covid-19-prostate-cross-tissue?scpbr=hca-covid-19-integrated-analysis | N/A |
|  | Nasal | Endothelial (0/134) | https://singlecell.broadinstitute.org/single_cell/study/SCP889/hca-lungmap-covid-19-barbry-nasal?scpbr=hca-covid-19-integrated-analysis | [45] |
|  | Brain | Endothelial (2/70) | https://singlecell.broadinstitute.org/single_cell/study/SCP891/hca-lungmap-covid-19-brain?scpbr=hca-covid-19-integrated-analysis | N/A |
|  | Bone marrow | N/A | https://singlecell.broadinstitute.org/single_cell/study/SCP895/hca-lungmap-covid-19-bone-marrow?scpbr=hca-covid-19-integrated-analysis | N/A |
|  | Skin cross tissue | EC vascular (0/1081)  EC lymphatic (0/437) | https://singlecell.broadinstitute.org/single_cell/study/SCP898/hca-lungmap-covid-19-skin-cross-tissue?scpbr=hca-covid-19-integrated-analysis | N/A |
|  | Skeletal muscle | EC vascular (0/1362)  EC lymphatic (0/644) | https://singlecell.broadinstitute.org/single_cell/study/SCP899/hca-lungmap-covid-19-skeletal-muscle-cross-tissue?scpbr=hca-covid-19-integrated-analysis | N/A |
|  | Breast tissue | EC vascular (0/1045)  EC lymphatic (0/164) | https://singlecell.broadinstitute.org/single_cell/study/SCP903/hca-lungmap-covid-19-breast-cross-tissue?scpbr=hca-covid-19-integrated-analysis | N/A |
|  | Colon | Endothelial (0/250) | https://singlecell.broadinstitute.org/single_cell/study/SCP902/hca-lungmap-covid-19-colon?scpbr=hca-covid-19-integrated-analysis | [58] |

**References**

45. Deprez, M., et al., *A single-cell atlas of the human healthy airways.* bioRxiv, 2019: p. 2019.12.21.884759.

46. Reyfman, P.A., et al., *Single-Cell Transcriptomic Analysis of Human Lung Provides Insights into the Pathobiology of Pulmonary Fibrosis.* Am J Respir Crit Care Med, 2019. **199**(12): p. 1517-1536.

47. Morse, C., et al., *Proliferating SPP1/MERTK-expressing macrophages in idiopathic pulmonary fibrosis.* Eur Respir J, 2019. **54**(2).

48. Habermann, A.C., et al., *Single-cell RNA-sequencing reveals profibrotic roles of distinct epithelial and mesenchymal lineages in pulmonary fibrosis.* bioRxiv, 2019: p. 753806.

49. Madissoon, E., et al., *scRNA-seq assessment of the human lung, spleen, and esophagus tissue stability after cold preservation.* Genome Biol, 2019. **21**(1): p. 1.

50. McDonough, J.E., et al., *Transcriptional regulatory model of fibrosis progression in the human lung.* JCI Insight, 2019. **4**(22).

51. Adams, T.S., et al., *Single-cell RNA-seq reveals ectopic and aberrant lung-resident cell populations in idiopathic pulmonary fibrosis.* Sci Adv, 2020. **6**(28): p. eaba1983.

52. Vieira Braga, F.A., et al., *A cellular census of human lungs identifies novel cell states in health and in asthma.* Nature Medicine, 2019. **25**(7): p. 1153-1163.

53. Lambrechts, D., et al., *Phenotype molding of stromal cells in the lung tumor microenvironment.* Nat Med, 2018. **24**(8): p. 1277-1289.

54. Goveia, J., et al., *An Integrated Gene Expression Landscape Profiling Approach to Identify Lung Tumor Endothelial Cell Heterogeneity and Angiogenic Candidates.* Cancer Cell, 2020. **37**(1): p. 21-36.e13.

55. Tucker, N.R., et al., *Myocyte Specific Upregulation of ACE2 in Cardiovascular Disease: Implications for SARS-CoV-2 mediated myocarditis.* medRxiv, 2020.

56. Ordovas-Montanes, J., et al., *Allergic inflammatory memory in human respiratory epithelial progenitor cells.* Nature, 2018. **560**(7720): p. 649-654.

57. Segerstolpe, Å., et al., *Single-Cell Transcriptome Profiling of Human Pancreatic Islets in Health and Type 2 Diabetes.* Cell Metab, 2016. **24**(4): p. 593-607.

58. Smillie, C.S., et al., *Intra- and Inter-cellular Rewiring of the Human Colon during Ulcerative Colitis.* Cell, 2019. **178**(3): p. 714-730.e22.
